# Center of Mass Compensation during Gait in Hip Arthroplasty Patients: Comparison between Large Diameter Head Total Hip Arthroplasty and Hip Resurfacing

**DOI:** 10.1155/2011/586412

**Published:** 2011-09-18

**Authors:** Vicky Bouffard, Julie Nantel, Marc Therrien, Pascal-André Vendittoli, Martin Lavigne, François Prince

**Affiliations:** ^1^Gait and Posture Laboratory, Marie Enfant Rehabilitation Center, Montreal, QC, Canada H1T 1C9; ^2^Department of Kinesiology, University of Montreal, Montreal, QC, Canada H3T 1J4; ^3^Orthopaedics Service, Division of Orthopaedic Surgery, Maisonneuve Rosemont Hospital, Montreal, QC, Canada H1T 2M4; ^4^Departement of Surgery, Faculty of Medicine, University of Montreal, Montreal, Qc, Canada H3T 1J4

## Abstract

*Objective*. To compare center of mass (COM) compensation in the frontal and sagittal plane during gait in patients with large diameter head total hip arthroplasty (LDH-THA) and hip resurfacing (HR). *Design*. Observational study. *Setting*. Outpatient biomechanical laboratory. *Participants*. Two groups of 12 patients with LDH-THA and HR recruited from a larger randomized study and 11 healthy controls. *Interventions*. Not applicable. *Main Outcome Measures*. To compare the distance between the hip prosthetic joint center (HPJC) and the COM. The ratio (R_HPJC-COM_) and the variability (CV_HPJC-COM_) were compared between groups. Hip flexor, abductor, and adductor muscle strength was also correlated between groups while radiographic measurements were correlated with the outcome measures. *Results*. In the frontal plane, HR shows less variability than healthy controls at push-off and toe-off and R_HPJC-COM_ is correlated with the muscle strength ratios (FR_ABD_) at heel contact, maximal weight acceptance, and mid stance. In the sagittal plane, LDH-THA has a higher R_HPJC-COM_ than healthy controls at push-off, and CV_HPJC-COM_ is significantly correlated with FR_FLEX_. *Conclusions*. One year after surgery, both groups of patients, LDH-THA and HR, demonstrate minor compensations at some specific instant of the gait cycle, in both frontal and sagittal planes. However, their locomotion pattern is similar to the healthy controls.

## 1. Introduction

Hip arthroplasty has become a standard procedure [1–3] to improve quality of life [4], restore physical capacities, relieve patients from pain [5, 6], and provide better hip function [5, 7] and stability [8]. Since younger patients are now more frequently affected by osteoarthritis (OA) [9], expectations of outcome after hip arthroplasty have changed. Indeed, patients not only want to get back to their daily living activities but also wish to return to a high level of physical activity [10], as soon as possible.

**Figure 1:**
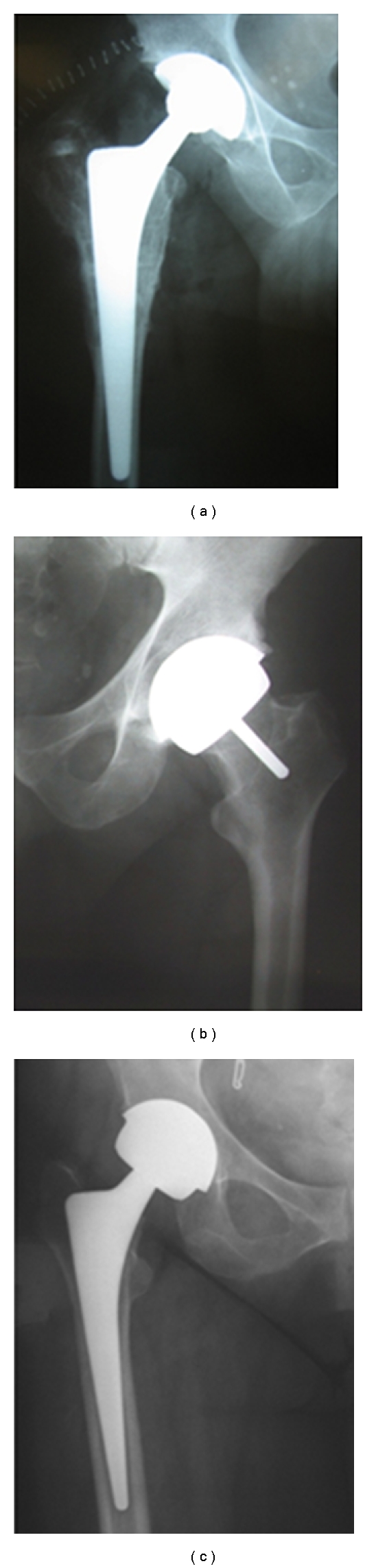
Prostheses evaluation in this study. (a) Total hip arthroplasty (THA), (b) surface replacement arthroplasty (HR), and (c) large diameter femoral head (LDH-THA).

The techniques available to treat the young patients with advanced OA, include, among others, total hip arthroplasty (THA) and hip resurfacing (HR). During THA procedure, a stem is inserted in the femoral canal while the femoral head and neck are resected and replaced by a femoral head of 28 mm diameter, articulating with a cup inserted in the acetabulum cavity (Figure 1(a)). Over the years, THA has proved its worthiness and is now recognized as an effective, reproducible and frequently used therapeutic option [11–13]. As for HR technique, the femoral head and acetabulum are shaped and covered with implants, using a large diameter femoral head size. This technique preserved more bone and became advantageous [13] (Figure 1(b)). More recently, a variation of THA has been used with a large diameter femoral head (LDH-THA) leaving the presence of a femoral stem the only anatomical difference between the two types of prosthesis (Figure 1(c)). Both types of implants, LDH-THA and HR, are felt to provide a better clinical function compared to THA [14, 15] because of the more anatomic femoral head that provides better hip range of motion and joint stability [16]. However, during total hip arthroplasty, THA and LDH-THA, the resection of bone and the insertion of a femoral stem make the reconstruction of hip biomechanical parameters hard to achieve [17]. The preservation of bone during HR leads to a better conservation of hip biomechanics and therefore of hip moments and muscular strength [18, 19]. This major difference between LDH-THA and HR may lead to impairment due to the type of prosthesis implanted.

After THA, pain is usually relieved and the range of motion improved, but normal activities may still be compromised in some patients [5]. An asymmetry in kinematics and kinetics during gait in patients with hip arthroplasty are to be expected [8]. Previous studies have reported that hip abductor muscles of patients undergoing THA generate less strength compared to those of healthy subjects [6, 20–24] and may explain the smaller hip abductor moment after THA [14] or limping during gait [5]. Furthermore, a decrease in hip abductor mechanical power [8] may also be responsible for trunk compensation [22] and abnormal gait pattern [14] in the frontal plane after THA. In the sagittal plane, a diminution in hip extension amplitude during late stance phase [25, 26], a decrease in the hip flexor and extensor moments [26] and a decrease in the work developed at the hip during push-off [8] can lead to gait impairment. Moreover, hip extensor muscle weakness may lead to disabilities during activities such as climbing stairs or rising from a chair [5]. The production of moment of force depends on muscle strength and moment arm lever. Patients undergoing hip arthroplasty have both these factors affecting the magnitude of the moment of force. Their muscle strength is modified by the preoperative conditions and postoperative rehabilitation while their moment arm lever is also modified by the surgery which may contribute to altered gait pattern in both frontal and sagittal planes. 

Even if patients recover mostly within the first three to six months after THA [4], complete recovery is likely never achieved [22] and patients have difficulty regaining normal walking patterns for several years after the surgery [6, 22, 24]. However, this new design of hip prosthesis may enhance gait pattern. Therefore, the purpose of this study is to compare COM position, with respect to HJC, during gait in frontal and sagittal plane in patients with LDH-THA and HR at 12 months after the operation.

## 2. Material and Methods

### 2.1. Patients

Patients with advanced hip joint degeneration were screened at the orthopaedic clinic of Maisonneuve-Rosemont Hospital, they were candidates for either LDH-THA or HR. Among volunteers recruited from a larger randomized study [27], patients with unilateral hip OA and who had no other lower limb affectations nor neuromuscular diseases that may interfere in their gait pattern, were selected for the study. A total of 35 individuals, divided into three groups (12 LDH-THA, 12 HR, and 11 healthy controls) participated in this study. Patients and external evaluators were blinded, with regards to type of arthroplasty, until one year after surgery. The healthy control subjects were recruited from the community through our institution. The project was approved by both the institutional research ethics and scientific committees. All participants were informed about the study and gave their written consent. 

All hip replacements were performed by three surgeons from the same hospital, and the posterior approach [28] was used for all interventions. During the HR procedures, a Durom hip resurfacing system with a large femoral head (Zimmer, USA) was used. For the LDH-THA group, CLS Sportono (Zimmer, USA) titanium uncemented prosthesis with a large femoral head was inserted. Restoration of biomechanics of the affected hip was performed based on preoperative template using the opposite side as a reference and by using intraoperative bony landmarks.

### 2.2. Radiographic Analysis

**Figure 2:**
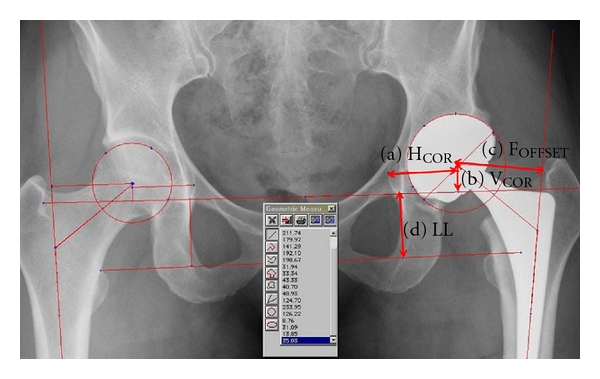
Radiographic measurement. (a) horizontal center of rotation (H_COR_), (b) vertical center of rotation (V_COR_), (c) femoral offset (F_OFFSET_), and (d) leg length (LL).

Standardized postoperative radiographs were taken. Anteroposterior radiographs of the pelvis were taken with the legs positioned at 15° of internal rotation. The radiographs were rejected if the coccyx was not centered on the pubic symphysis and located proximally within 2 to 4 cm to ensure proper positioning of the pelvis in both frontal and sagittal planes. The radiographs were scanned (VIDAR VXR-12, USA) and analysed using Imagika software (Clinical Measurement Corporation, USA). The horizontal (H_COR_) and vertical (V_COR_) center of rotation of the prosthetic hip joint, the femoral offset (F_OFFSET_), and leg length (LL) inequality were measured for the replaced and contralateral hip (Figure 2) [29]. The H_COR_ is the distance between the V_COR_ line and the radiographic teardrop. The V_COR_ is defined by the perpendicular distance from the center of rotation of the hip to the inter teardrop line. The F_OFFSET_ is the length of a line connecting the hip joint center and perpendicular to an extension of the femoral shaft line. Finally, the LL is the length of a line perpendicular and connecting to the interteardrops line and the interlesser trochanter line. All distances were measured in mm.

### 2.3. Tasks

Subjects were asked to walk at their normal speed on a 10-meter walkway with two embedded force platforms (Advanced Mechanical Technology Inc., USA) recording at 120 Hz. Three gait cycles were collected for each subject, 12 months postoperatively. Trials were included when both feet made full contact on each of the two force platforms. A sufficient resting period was given between trials to avoid fatigue. Subjects were tested barefoot, wearing shorts, and t-shirt. Twenty-nine 14 mm diameter reflective markers were positioned on bony landmarks to define body segments using Vicon Plug- in-Gait model [30]. Spatiotemporals, kinetics and kinematics were recorded, at 60 Hz, by an 8-camera Vicon system (Oxford Metrics Limited, UK). 

**Figure 3:**
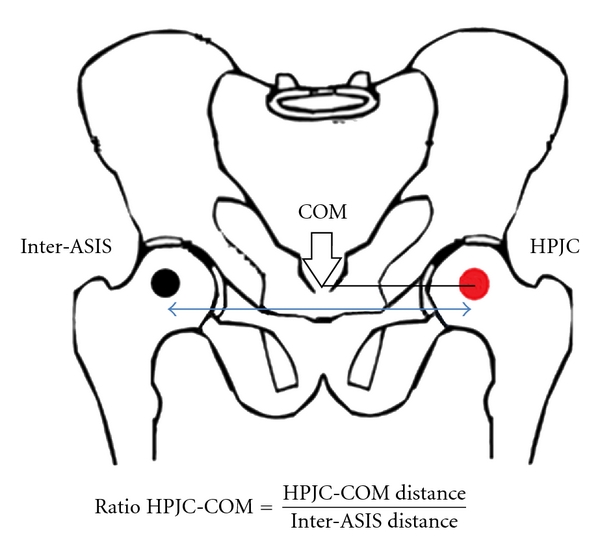
Illustration for the R_HPJC-COM_ in the frontal plane.

**Figure 4:**
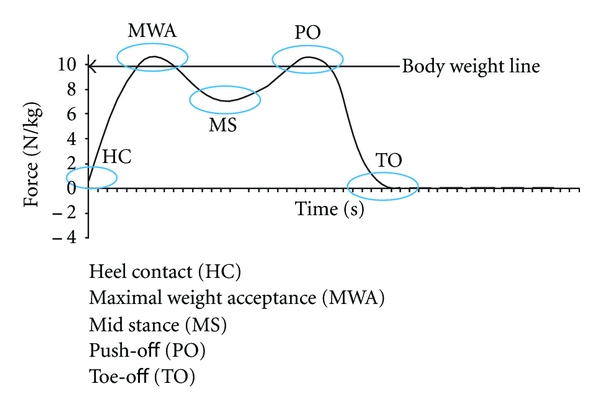
Vertical ground reaction forces during normal gait. Heel contact (HC), maximal weight acceptance (MWA), mid stance (MS), Push-off (PO), and Toe off (TO).

The COM position was calculated from marker positions and anthropometric tables [31] while the distance (in mm) between HPJC and COM was calculated in both frontal and sagittal planes. Distances were normalized by the interanterior superior iliac spine (ASIS) distance which was measured with the Vicon workstation software from markers positions. For each individual, the ratio (R_HPJC-COM_) was determined as the distance between HPJC-COM/inter-ASIS distance (Figure 3) for both the frontal and sagittal planes. The coefficient of variation (CV_HPJC-COM_) of the distance between HPJC and COM was also calculated [32]. The method used to calculate the CV_HPJC-COM_ was

(1) $${\text{CV}}_{\text{HPJC-COM}}=\left|\frac{\sigma }{\mu }\right|*100,$$

where *σ* is the standard deviation and *μ* the mean of the HPJC-COM distance. Data were calculated at five specific instants of the gait cycle, extracted from vertical ground reaction forces: (1) heel contact, (2) maximum weight acceptance, (3) midstance, (4) push-off, and (5) toe-off (Figure 4).

Hip abductor, adductor, and flexor muscle strength of both sides was tested. For the hip flexor muscle, the patient was seated [33] while for the hip abductor and adductor muscle the patient was lying on an examination table [34]. A Penny and Giles hand-held dynamometer (Penny and Giles, UK) was used. The test was repeated twice for each muscle with a resting period of a minute. The average peak force generated in Newtons was expressed as the % of the peak force generated of the sound limb and for the healthy controls, the left limb was expressed relative to the right limb (FR_ABD_, FR_ADD_, and FR_FLEX_). 

### 2.4. Statistical Analysis

All statistical analyses were performed using SPSS 17.0 (SPSS Inc., USA). The sociodemographic (age, weight, height, and BMI) and spatiotemporal data were analysed using one-way analysis of variance (ANOVA). Chi-square test was used for gender. The R_HPJC-COM_ and CV_HPJC-COM_ were analyzed using a one-way ANOVA. The results were then further analyzed if necessary, with Tukey post hoc test. For non-normality distributed data, as required, results were analyzed with a Kruskal-Wallis test. In the frontal plane, Pearson correlations were calculated between the parameters evaluated R_HPJC-COM_ and CV_HPJC-COM_ and the F_OFFSET_, H_COR_, FR_ABD_, and FR_ADD_, while in the sagittal plane, correlations were made between R_HPJC-COM_ and CV_HPJC-COM_ and V_COR_, LL, and FR_FLEX_. Correlations included both groups, LDH-THA and HR. Significant difference was set at *P* < 0.05.

## 3. Results

No difference was observed between the three groups for the sociodemographic data (Table 1).

Statistical difference was found for the spatiotemporal data (Table 2). The healthy controls showed a significantly slower cadence compared to LDH-THA (*P* = 0.00) and HR (*P* = 0.04) group. Healthy controls also showed a reduced walking speed compared to LDH-THA (*P* = 0.02) group.

### 3.1. Frontal Plane Analysis

**Figure 5:**
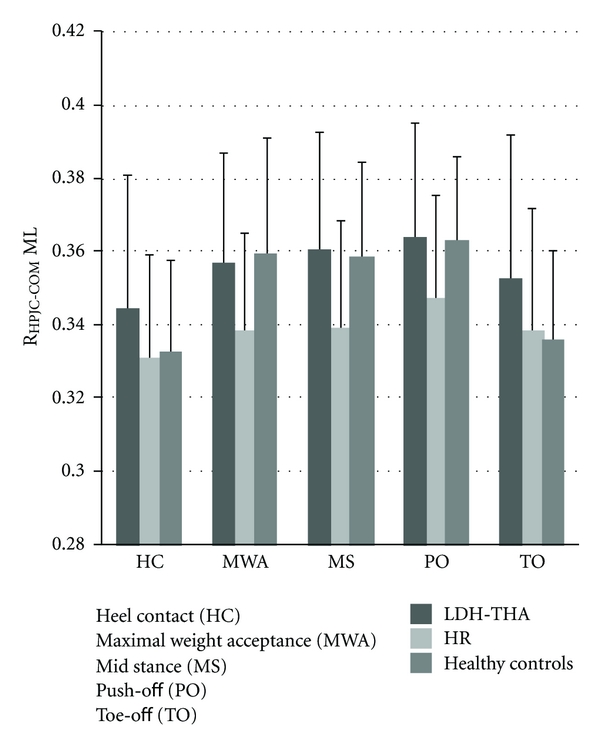
Mean R_HPJC-COM_ in the frontal plane for LDH-THA, HR, and healthy controls at five instants of the gait cycle.

No statistical difference was found for the R_HPJC-COM_ between the three groups (Figure 5).

**Figure 6:**
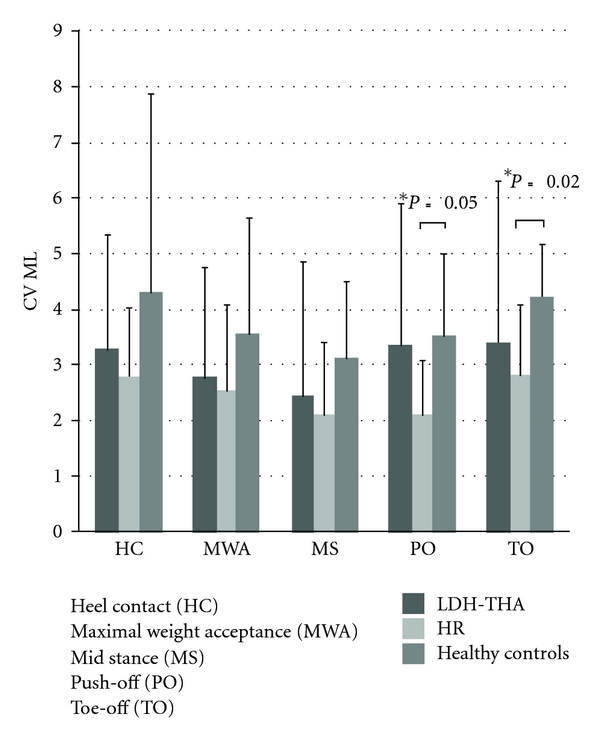
Mean CV_HPJC-COM_ in the frontal plane for LDH-THA, HR, and healthy controls at five instants of the gait cycle.

A statistical difference was found for the CV_HPJC-COM_ in HR patients compared to healthy controls at push-off (*P* = 0.05) and toe-off (*P* = 0.02). Patients undergoing HR tended to show less variability than healthy controls (Figure 6).

### 3.2. Sagittal Plane

**Figure 7:**
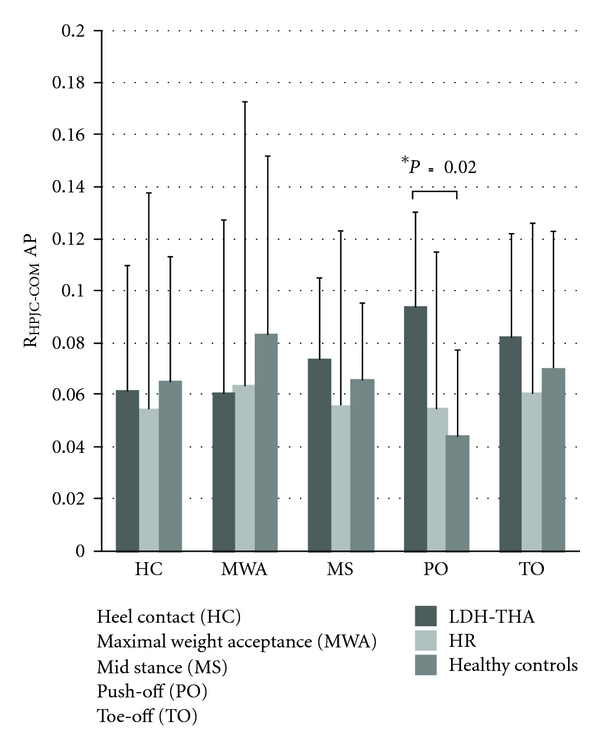
Mean R_HPJC-COM_ in the sagittal plane for LDH-THA, HR, and healthy controls at five instants of the gait cycle.

A statistical difference was found for the R_HPJC-COM_ for the LDH-THA patients compared to healthy controls at push-off (*P* = 0.02). LDH-THA patients seem to increase their HPJC-COM moment lever arm by bending their trunk forward (Figure 7).

**Figure 8:**
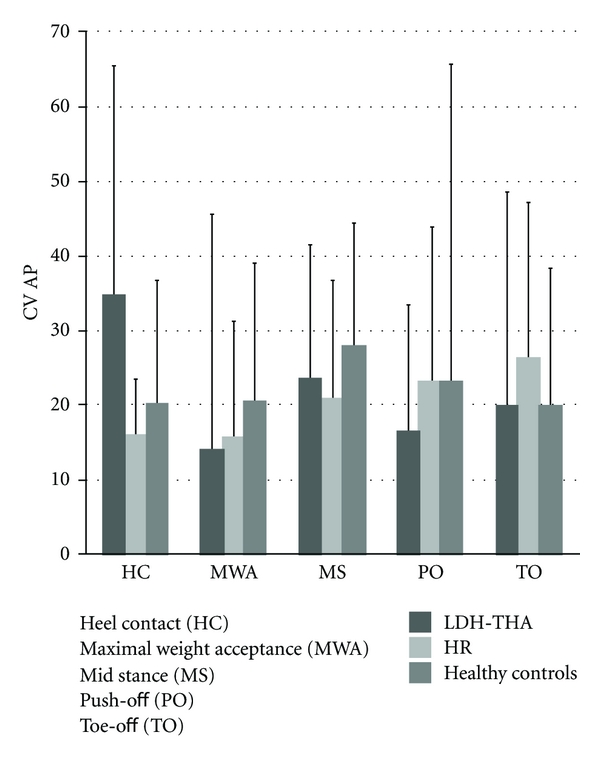
Mean CV_HPJC-COM_ in the sagittal plane for LDH-THA, HR, and healthy controls at five instants of the gait cycle.

No statistical difference was found between the three groups for the CV_HPJC-COM_ (Figure 8).

### 3.3. Correlations

In the frontal plane, no correlation was found between the R_HPJC-COM_ and the radiographic measurements (F_OFFSET_, H_COR_). On the other hand, correlations were found between the R_HPJC-COM_ and the FR_ABD_ at heel contact (*P* = 0.04), maximum weight acceptance (*P* = 0.04) and mid stance (*P* = 0.02). No correlation was found for the CV_HPJC-COM_ (Table 3). In the sagittal plane, no correlation was found for the R_HPJC-COM_ and the radiographic measurements (V_COR_, LL) and the force ratio (FR_FLEX_). However, a correlation was found for the CV_HPJC-COM_ and the FR_FLEX_ at MWA (*P* = 0.00) (Table 4).

## 4. Discussion

Lavigne et al. [27], using different outcome measures such as questionnaires (WOMAC, SF-36, Merle D'Aubigné, and UCLA), postural balance tests (total path length of center of pressure), spatiotemporal analysis (velocity, cadence, and step length), and functional tests (functional reach, time up and go hip flexors and abductors strength, step, and hop tests) were not able to demonstrate a clinical benefit of HR over LDH-THA.

Using a selected subgroup of patients from this study, the aim of the present study was to determine if the presence of a femoral stem combined to the loss of bone in LDH-THA have an impact on COM position, with respect to HJC, during gait at 12 months after surgery compared to HR. Data from LDH-THA and HR patients were also compared to a healthy controls group. 

The statistical analysis showed no difference in sociodemographic data while a statistical difference was found in some spatiotemporal parameters. Particularly, LDH-THA and HR patients of this study showed a higher cadence compared to healthy controls while LDH-THA patients walked with a greater velocity when compared to healthy controls. In previous studies, patients undergoing THA were walking slower than healthy controls [8, 14, 22, 35]. Other studies, which compared THA and HR, showed that THA were walking slower compared to HR [14, 36]. In the present study, the more anatomical head used in LDH-THA patients' might have improved biomechanical reconstruction that provides better stability and hip range of motion and explains the faster walking velocity found in this group [27]. The indications for the walking task were the same for all subjects. Trials were done at a self-selected speed. After LDH-THA and HR, patients might want to perform during gait experimentation to prove they have completely recovered from their surgical intervention.

### 4.1. *R*_HPJC-COM_ and *CV*_HPJC-COM_ in the Frontal Plane

In order to maintain pelvic equilibrium, agonist and antagonist muscles must generate an equal net moment [37]. One way to compensate for weaker muscle strength may be to shift the body COM toward the hip prosthesis joint center. This strategy implies a reduction of the moment arm lever, which consequently causes a decrease in the magnitude of the hip muscle strength [23] needed to ensure pelvis stability. Contrariwise, an increase of the muscular strength is needed to maintain the pelvic in equilibrium when a longer moment arm lever is created. This higher muscular demand leads to muscular fatigue that could impair the gait pattern compared to the healthy controls. 

During walking, the proper function of hip muscles is mandatory to maintain stability of the head-arm-trunk (HAT) segment. In the frontal plane, this role is achieved by the abductor muscles [31]. Although, hip arthroplasty has become one of the most successful orthopaedic procedures [38–40], impairments such as abductor muscles weakness [22, 23, 41] may persist after THA. This impairment could interfere during gait and lead to trunk compensations [22, 24, 42] in the frontal plane [14, 22]. In the present study, no statistical difference was found between the groups for the R_HPJC-COM_ in the frontal plane. However, HR patients maintain a smaller R_HPJC-COM_ than LDH-THA and healthy controls on almost all the gait cycle. This suggests that LDH-THA and HR patients performed as well as healthy controls but used different strategies. In fact, patients undergoing LDH-THA did not decrease their HPJC-COM moment arm length by shifting body weight toward the affected limb in order to reduce constraints on their prosthetic hip joint and minimize the effort of hip abductor muscles [43]. Patients undergoing HR reduce muscles strain of the hip abductors by positioning their COM to create a mechanical advantage. These results are in accordance with the literature. In their study, comparing conventional THA and HR, Nantel et al. [15] found that, patients undergoing THA showed lower abductor energy generation at the end of the stance phase compared to healthy controls. This difference was explained by hip abductor weakness of the operated side compared to the contralateral leg within six to eight months postoperatively. Moreover, according to Nantel et al. [15] hip power of the HR patients, in the frontal plane, is lower than LDH-THA and healthy controls during the totality of the gait cycle. The absence of difference between LDH-THA and HR could be explained by the use of the large diameter head in THA. According to Lavigne et al. [27] LDH-THA promotes a better biomechanical reconstruction than THA and may challenge the superior clinical outcomes of HR [44–46]. Furthermore, in our study, gait analyses were performed one year postoperatively which have left more time for patients to recover from their surgery. Asayama et al. [23] suggested that a strength ratio (operated limb/nonoperated limb) near 88% is the threshold below which functional manifestation of abductor weakness starts to appear while compensation during gait, for example, limping or delayed Trendelenburg test, may appear when the strength ratio is around 72%. In other words, patients who underwent hip arthroplasty may have not recovered their full hip abductor strength but compensation cannot be seen unless the force ratio is less than 72%. One year postoperatively patients who underwent hip arthroplasty, in this study, are able to control their COM position as the healthy controls group but LDH-THA and HR use different strategies. 

It has already been established that identical movement patterns cannot be generated by successive attempts [47]. In other words, the role that variability plays in coordination and control of the sensorimotor system is a central issue for the study of motor control [48]. Without minimal steadiness of the locomotor pattern, humans cannot master modulations [49] and may adapt their gait pattern in order to overcome this impairment. 

In our study, a statistical difference was found for the CV_HPJC-COM_ between HR and healthy controls at the end of the stance phase (push-off and toe-off) but HR and LDH-THA are less variable than healthy controls for all the gait cycle. These results might be explained by the surgical intervention. Normally, hip joint stability is partly provided by the strong ligaments and powerful muscles [50] surrounding the hip articulation. During the posterior surgical approach, several muscles, tendons, and ligaments are affected by the surgical technique and may compromise hip joint stability. After the surgery, LDH-THA and HR patients of the present study showed less variability for the COM position relative to the HPJC and statistically significant for the HR at the end of the stance phase compared to healthy controls. Patients with LDH-THA and HR might adapt their gait pattern in order to increase hip joint stability for compensating the weaker structures surrounding the hip joint. After surgery, hip arthroplasty patients may not be confident in recovering from a large excursion of their COM in the frontal plane while the healthy controls have the ability to recover from these situations. HR patients position their COM at the same place, in order to enhance better propulsion and to promote a safe swing phase. The presence of the same pattern between the two type of prosthesis, LDH-THA and HR, suggests that adaptations of the gait pattern might arise from the surgical procedure.

### 4.2. *R*_HPJC-COM_ and *CV*_HPJC-COM_ in the Sagittal Plane

In the sagittal plane, the effects of surgery may also have an impact on the patient's gait strategies mainly on the HAT segment that is under the control of the hip flexor and extensor muscles [51]. In the present study, a statistical difference was found for the R_HPJC-COM_ at push-off between LDH-THA and healthy controls. Specifically, LDH-THA patients showed an increased R_HPJC-COM_ distance, which increased the moment lever arm at PO. This can be done by bending the trunk forward which creates a mechanical advantage and improves the extensor moment at the hip. Previous studies [22, 41] have shown that patients undergoing conventional THA have to restrain their hip extension excursion at the end of the stance phase in order to compensate for hip flexor contractures compared to HR patients. In a recent study, Lavigne et al. [27] concluded that LDH-THA have a better hip range of motion compared to HR and THA. The femoral stem combined with the large femoral head size in LDH-THA provide better hip range of motion; however, they did not reach normal hip motion. Modifications of the gait pattern after surgery might be a compensation for a reduced range of motion due to pain in OA patients while postsurgery adaptations can be due to fear of pain [52]. In the present study, LDH-THA patients may have been more affected pre- and postoperatively.

### 4.3. Correlations

During gait, contribution from the hip abductor muscles is more important from heel contact to mid stance because they help in supporting the body weight. To reduce the muscular demand on hip muscles patients must bring their COM closer to the HJC. In the present study, a negative moderate [53] correlation was found between R_HPJC-COM_ and the FR_ABD_ from heel contact to mid stance. These results suggest that stronger they are, patients undergoing hip arthroplasty can more easily position their COM closer to their HJC while patients who are weaker cannot reduce their moment lever arm.

In the sagittal plane, muscle involvement is also essential in order to maintain pelvis stability. The results showed a moderate positive correlation between the CV_HPJC-COM_ and the FR_FLEX_ at maximum weight acceptance (*P* = 0.04). These results propose that when patients undergoing hip arthroplasty are stronger, they can afford to be more variable because they will be able to manage excursion of their COM more easily at that instant.

### 4.4. Limits

In the present study, the predictive method [54] was used to calculate the hip joint center (HJC). This approach calculates the three dimensional coordinates of HJC from linear regressions and the size of different body segments. For example, some models use the width, length and depth of the pelvis to determine the HJC. This approach does not account for differences between individuals. Recently, a new method known as the functional method was developed [55]. This method use the geometry of hip movements in its three degrees of freedom (flexion-extension, abduction-adduction, and rotation) [55] to determine more precisely the emplacement of the HJC. The latter method may have been more efficient in this study because it would take into consideration the individual characteristics of patients who underwent hip surgery and it could have a major impact on the results. Moreover, the analyses were done one year after the surgery when the recovery mostly takes place within the first to 3 to 6 months after the operation [4].

## 5. Conclusion

One year after surgery, patients undergoing LDH-THA or HR still have gait impairments, at some instants during the gait cycle, related to the positioning and variability of their COM with respect to their hip joint center in both the frontal and sagittal planes compared to healthy controls. The use of a large diameter head in THA seems to reduce the anatomical difference with HR and no major differences were found during locomotion between these two types of prostheses and they walked like healthy controls. However, walking is a simple task compared to physical activities such as sports. The integration of a specific rehabilitation program would be necessary to promote their participation in sports and prepare them for higher demanding activities.

## Figures and Tables

**Table 1:** Mean (SD) of the sociodemographic data.

	Age (y)	Gender	Height (m)	Weight (kg)	BMI (kg/m^2^)
LDH-THA	50.8 (6.1)	7 M/5 F	1.68 (0.04)	75.3 (15.3)	26.7 (4.7)
HR	52.8 (6.7)	6 M/6 F	1.67 (0.08)	74.1 (15.4)	26.3 (3.8)
Healthy controls	45.7 (8.2)	4 M/7 F	1.67 (0.09)	73.5 (11.3)	26.3 (3.0)

**Table 2:** Mean (SD) of the spatiotemporal data.

	Cadence (step/min)	Velocity (m/s)	Step length (m)
LDH-THA	128.9 (8.3)	1.51 (0.20)	1.40 (0.13)
HR	125.2 (7.3)	1.41 (0.17)	1.35 (0.13)
Healthy controls	117.0** (7.4)	1.31* (0.15)	1.34 (0.13)

*Statistical significance between healthy controls and LDH-THA.

**Statistical significance between healthy controls and both LDH-THA and HR.

**Table 3:** Correlations between the R_HPJC-COM_ and the force ratio in the frontal plane.

	*FR*_*ADD*_		*FR*_*ABD*_	
	Pearson coefficient	*P*	Person coefficient	*P*
HC	0.21	0.36	−0.46	0.04*
MWA	0.15	0.53	−0.46	0.04*
MS	0.04	0.86	−0.50	0.02*
PO	0.16	0.53	−0.36	0.11
TO	0.13	0.58	−0.30	0.19

*Statistical significance.

**Table 4:** Correlations between the CV_HJPC-COM_ and the force ratio in the sagittal plane.

	FR_FLEX_	
	Pearson coefficient	*P*
HC	−0.31	0.18
MWA	0.60	0.00*
MS	0.09	0.71
PO	−0.19	0.40
*TO*	−0.37	0.10

*Statistical significance.
